# Physical Characteristics Associated With a History of Ankle Sprain in Elementary School Badminton Players

**DOI:** 10.7759/cureus.97498

**Published:** 2025-11-22

**Authors:** Masashi Matsumura, Yasushi Kurihara

**Affiliations:** 1 Physical Therapy, Department of Rehabilitation, Kyorin University, Faculty of Health Sciences, Tokyo, JPN; 2 Department of Physical Therapy, Josai International University, Faculty of Social Work Studies, Togane, JPN

**Keywords:** ankle flexibility, ankle sprain, badminton, elementary school student, knee extension

## Abstract

Background and objective

Ankle injuries are prevalent among elementary school students and are associated with subsequent injuries. This study aimed to investigate the relationship between a history of ankle sprain and the physical characteristics of elementary school badminton players.

Methods

A questionnaire-based survey was conducted among 64 fifth- and sixth-grade badminton players. Based on the presence or absence of a history of ankle sprain, the participants were categorized into the sprain and non-sprain groups. Both groups underwent assessments of dynamic balance ability, flexibility, muscle strength, and foot evaluation. Players whose sprained sides matched their dominant foot were included in the analysis.

Results

A total of 15 players were assigned to the sprain group and 49 to the non-sprain group. In the sprain group, 11 players had their sprained side matched to their dominant foot. The sprain group showed a significantly higher proportion of patients who were unable to squat, a significantly stronger knee extension strength, and a significantly lower ankle dorsiflexion range of motion.

Conclusions

A history of ankle sprain in elementary school badminton players is associated with ankle flexibility and knee extension strength. In badminton, repetitive lunging and jumping movements pose a high risk of ankle sprain, which is the most common type of lower limb injury. Limited ankle dorsiflexion range of motion (ROM) has been identified as a characteristic feature of the sprain group, suggesting that it is a critical factor requiring particular attention after an ankle sprain.

## Introduction

Badminton is one of the most popular sports worldwide. According to the Badminton World Federation, more than 300 million people actively participate in the sport globally [1]. In Japan, the Nippon Badminton Association reported that of the 291,983 registered members in 2023, 21,787 were elementary school students aged 7-12 years [2]. In addition, elite badminton players in Malaysia usually begin training as early as the age of 9-10 years [3]. These figures highlight badminton’s large global participant base and the early age at which many players start the sport.

Badminton is a physically and technically demanding sport that requires jumping skills, speed, strength, accuracy, and agility; consequently, it is associated with a high incidence of injuries. Common injuries include knee ligament damage, ankle sprains, lower back problems, shoulder injuries, and various overuse conditions, with many occurring more often during training than in competition [4-8]. However, at the elite level, injuries are reported to occur more frequently in matches than in practice [9,10]. Among sports-related ankle injuries, sprains are the most prevalent [11], and in badminton specifically, sprains represent the most common injury type, accounting for 36.06% of all cases [12]. Other ankle joint-related injuries in badminton include Achilles tendon ruptures, which occur in 2.6-5.3% of cases [13,14]. Therefore, badminton has a high incidence of ankle sprains among lower limb injuries. In badminton, players must perform rapid lunges and jumps in multiple directions in response to their opponents’ movements and quickly return to a central position to prepare for the next move [15,16]. Consequently, the ankle is one of the most susceptible areas to injury.

Acute lateral ankle sprains (LASs) are frequent injuries in sports, including badminton [17-19]. Mechanically, LASs can result from both non-contact and contact events, typically involving internal rotation of the ankle joint followed by plantar flexion [20]. Moreover, 95% of ankle sprains are caused by non-contact injuries [20]. In badminton, a forefoot landing posture with a plantarflexed and internally rotated ankle joint configuration can cause LAS [17]. Patients with LAS report a decrease in the range of motion (ROM) of the ankle joint, continuous pain, muscle weakness, feelings of weakness, and decreased function [18,21,22]. Residual problems after sprains include pain (30.2%), instability (20.4%), crepitus (18.3%), muscle weakness (16.5%), stiffness (14.6%), and swelling (13.9%) [23]. In basketball, dorsiflexion and plantar flexion ROM of the ankle joint on the side with a history of sprains is reduced, and a decrease in dorsiflexion and plantar flexion muscle strength is observed [24]. A cohort study of female soccer players reported that those with ankle sprains demonstrated poorer performance in both double-leg and single-leg balance tests compared to those without ankle sprains.

Additionally, players with a lower hamstrings-to-quadriceps muscle strength ratio are significantly more prone to developing sprains [25]. The predictors of balance in patients with functional ankle instability include ankle plantar flexion strength, dorsiflexion/plantar flexion ROM, and pain [26]. Dorsiflexion ROM, plantar cutaneous sensation, eversion muscle strength, and static balance are important for dynamic balance function in chronic ankle instability [27]. Therefore, sprains can lead to various dysfunctions and negative effects even after the injury has healed. However, these outcomes have not been specifically studied in badminton players.

Our investigation of injury prevalence among elementary school badminton players revealed that ankle injuries were the most common [28]. Other studies focusing on Japanese elementary school badminton players have reported that 71 of 478 participants sustained ankle injuries, 74 had knee injuries, and 48 experienced shoulder injuries. Furthermore, among players with a history of ankle injury, 21.1% re-injured their ankle, 28.4% subsequently sustained a knee injury, and 37.5% later suffered a shoulder injury [29]. These findings indicate that ankle injuries are prevalent even among elementary school students and are associated with subsequent injuries. In the present study, we aimed to examine the differences in ROM, muscle strength, dynamic balance, and foot arch between elementary school badminton players with and without a history of ankle sprain.

## Materials and methods

Participants

Sixty-four fifth- and sixth-grade badminton players aged 10-12 years, registered in 20 club teams under the Japan Schoolchildren Badminton Federation, were included in this study. Data were collected between May and August 2022. After obtaining approval from the club team representatives, the study procedures were explained to the players, and informed consent was obtained. The inclusion criteria were as follows: no current pain or physical limitations, the ability to perform at their usual competitive level, and the presence of a parent or guardian at the time consent was given. Participants were excluded if they had any orthopedic conditions that could influence the measurements.

Methods

The study employed a cross-sectional design. Before the measurements, a questionnaire was distributed to the participants. The survey items included age, sex, height, weight, years of experience playing badminton, dominant hand and foot, and history of sports-related injuries. The dominant hand was defined as the hand that gripped the racket, and the dominant foot was defined as the foot used to kick the ball. For those who responded “Yes” to having a history of injury, additional questions were asked regarding the presence of current injuries, timing of the injuries, and injury sites (multiple responses were allowed). Sports injury history was defined as injuries sustained during badminton competitions within the past year resulting in one or more of the following: 1) immediate cessation of playing badminton, 2) absence from the next scheduled competition, or 3) consultation with a physician [29]. The experience of an ankle sprain was evaluated in all participants, regardless of the time of injury. Based on the results of the questionnaire survey, the participants were divided into two groups: the sprain (those with a history of an ankle sprain) and the non-sprain (those without a history of an ankle sprain) groups.

The measurement items included dynamic balance, flexibility, muscle strength, and foot evaluation. The Modified Star Excursion Balance Test (mSEBT) was used to assess dynamic balance. Flexibility was assessed based on the ability to squat, stand forward flexion, heel-to-toe distance, and ankle dorsiflexion ROM. Lower limb muscle strength (hip abduction, knee extension, and toe grip) was measured. Foot evaluation involved measuring the foot-to-arch height ratio. All measurements were conducted by a licensed physical therapist in a meeting room with a hard floor located in the gymnasium. Each participant wore the shoes they typically used for badminton, and measurements were performed between 10:00 and 16:00. The examiner was blinded to whether each participant had a history of ankle sprain.

**Figure 1:**
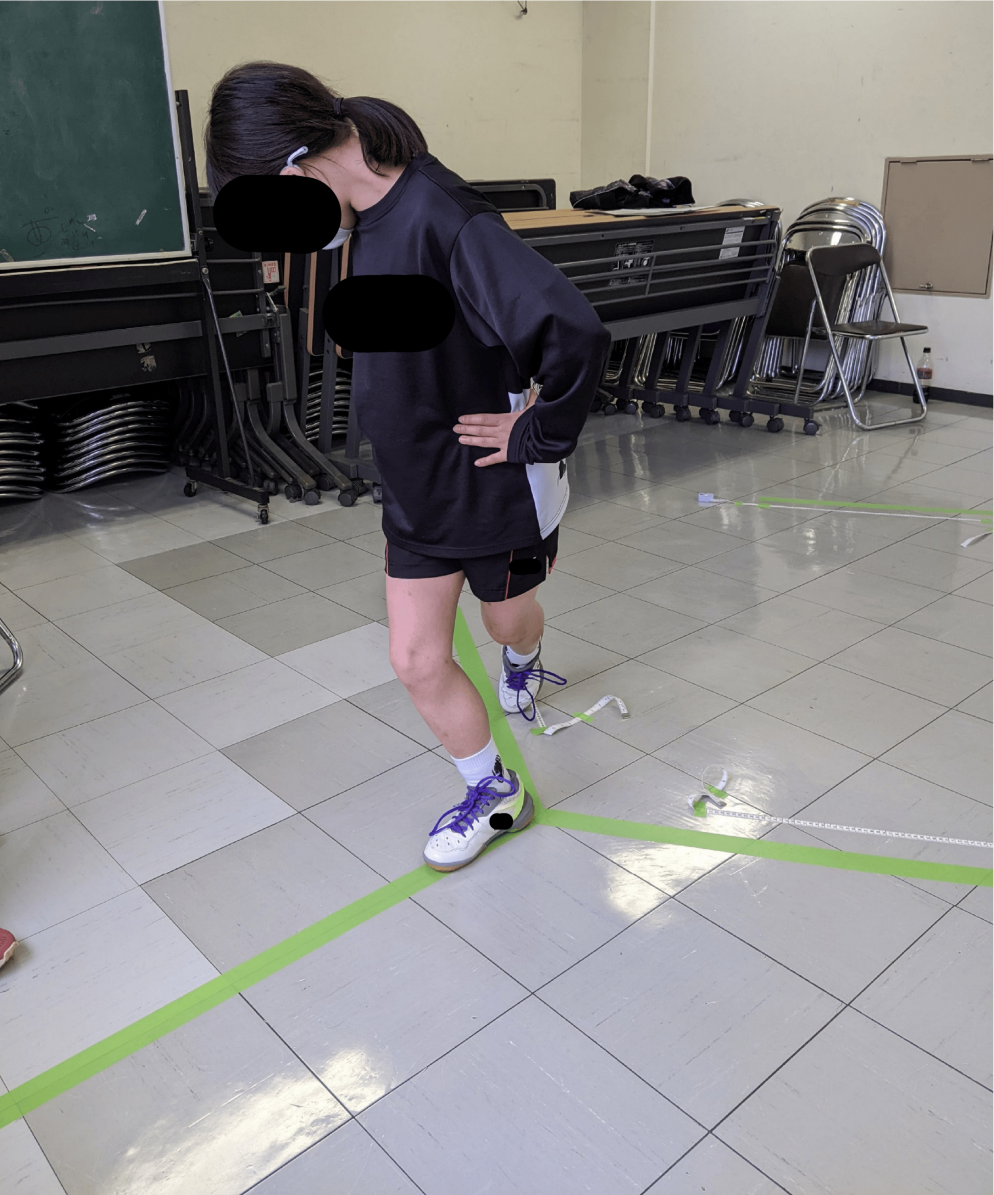
The Modified Star Excursion Balance Test (mSEBT)

The mSEBT was conducted using the method proposed by Plisky et al. (Figure 1) [30]. The measurements were performed by two examiners who had a thorough understanding of the mSEBT. A central point was established on the floor, and using a tape, straight lines were drawn from this point in three directions: forward, backward-inward, and backward-outward. The participants stood with their hands at both sides of their hips, balanced on one leg at the center of the lines, and reached with the opposite leg along each line as far as possible. Participants practiced to enable performance of the task without compensation in each direction; thereafter, two measurements for each direction were performed, and the average value was recorded. The measurements were performed by alternating the supporting legs. The reach distances in the mSEBT were normalized to the limb lengths of the participants, and the average values for the three directions were calculated [31].

**Figure 2:**
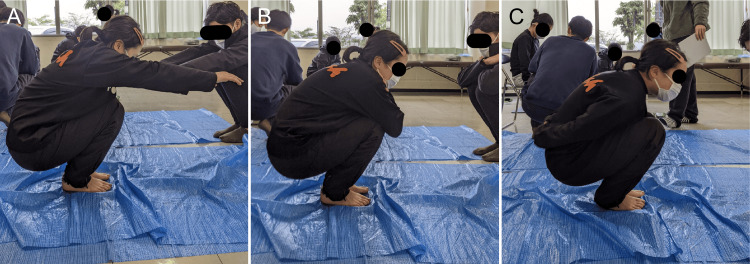
Squat A. Keeping the arms forward. B. Crossing the arms in front of the chest. C. Clasping the hands at the waist

Squat ability was assessed using the following procedures. At the starting position, the participant stood barefoot with both feet together, placing the inner sides of the feet and knees together, and keeping the knees extended. From this starting position, the participant slowly squatted until the buttocks and heels were as close together as possible, which was defined as the ending position. The measurements were performed in three patterns: 1) keeping the arms forward (shoulder flexed at 90°); 2) crossing the arms in front of the chest; and 3) clasping the hands at the waist. The tasks were performed in order, starting from 1. In any of the patterns, the measurement was stopped if the participant could not hold the ending position for two seconds, if their knees moved apart during the squatting motion, or if their heels were lifted off the ground (Figure 2).

**Figure 3:**
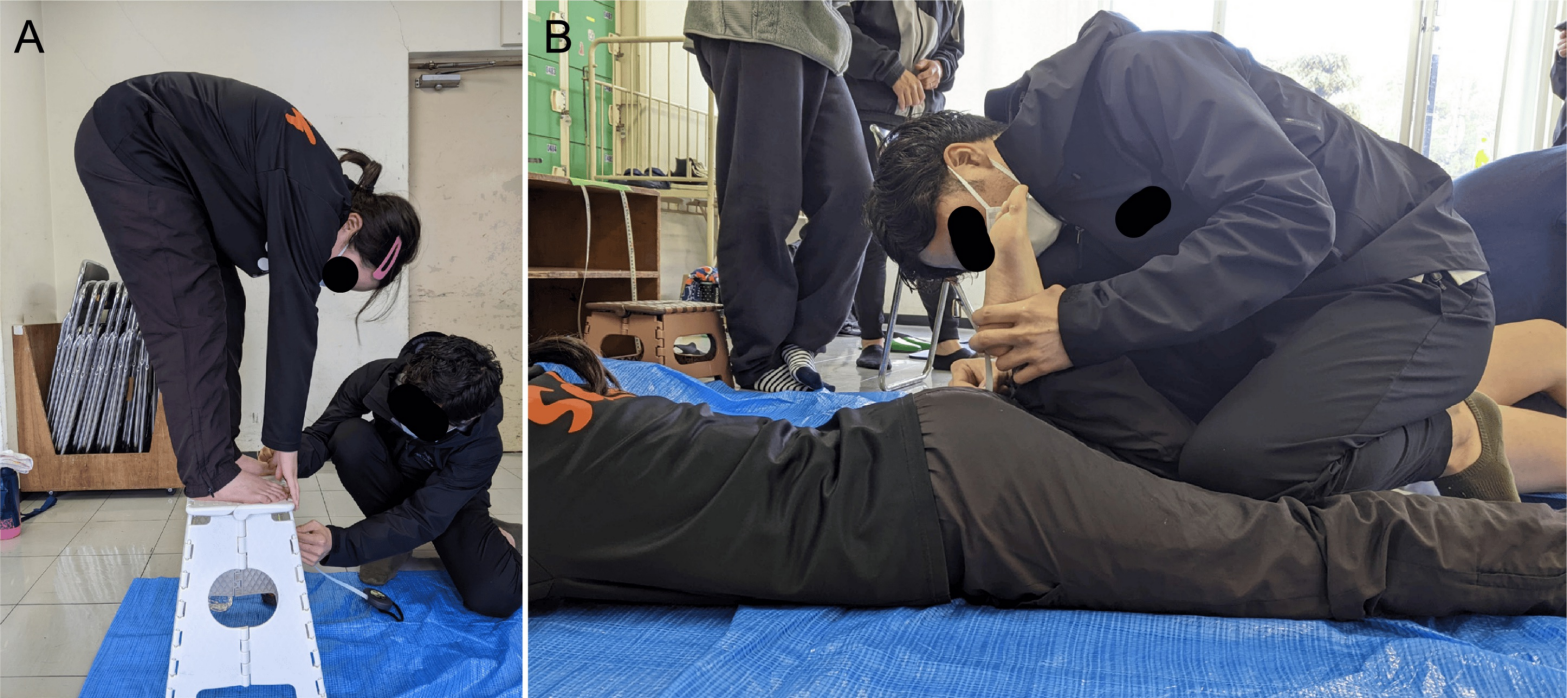
Measurements of flexibility A. Standing forward flexion. B. Heel-to-buttock distance

Standing forward flexion was measured using a digital forward flexion meter (model Flexion-D, Takei, Japan). Measurement of limb position involved placing both heels together and spreading the toes approximately 5 cm apart and aligning them with the front edge of the platform, while keeping the knees extended. The participants were instructed to bend forward without bending their knees using a smooth motion, and the distance bent forward was measured by pressing the tip of their fingers against a digital forward flexion meter; they were instructed not to perform the measurement using only one arm. Considering the impact on the results, the participants performed the measurement once after the researcher demonstrated the task (Figure 3A). Heel-to-buttock distance was measured. In the prone position, the knee joint on the measurement side was passively flexed, and the distance from the heel to the buttock was measured using a tape measure. If the buttocks were lifted off the surface as a compensatory motion, the measurement was performed at the maximum position without compensation (Figure 3B). Ankle dorsiflexion ROM was assessed in the supine position with knee flexion. A goniometer (Tokyo University style) was used to measure the angle formed by a vertical line to the fibula and fifth metatarsal during passive movement.

**Figure 4:**
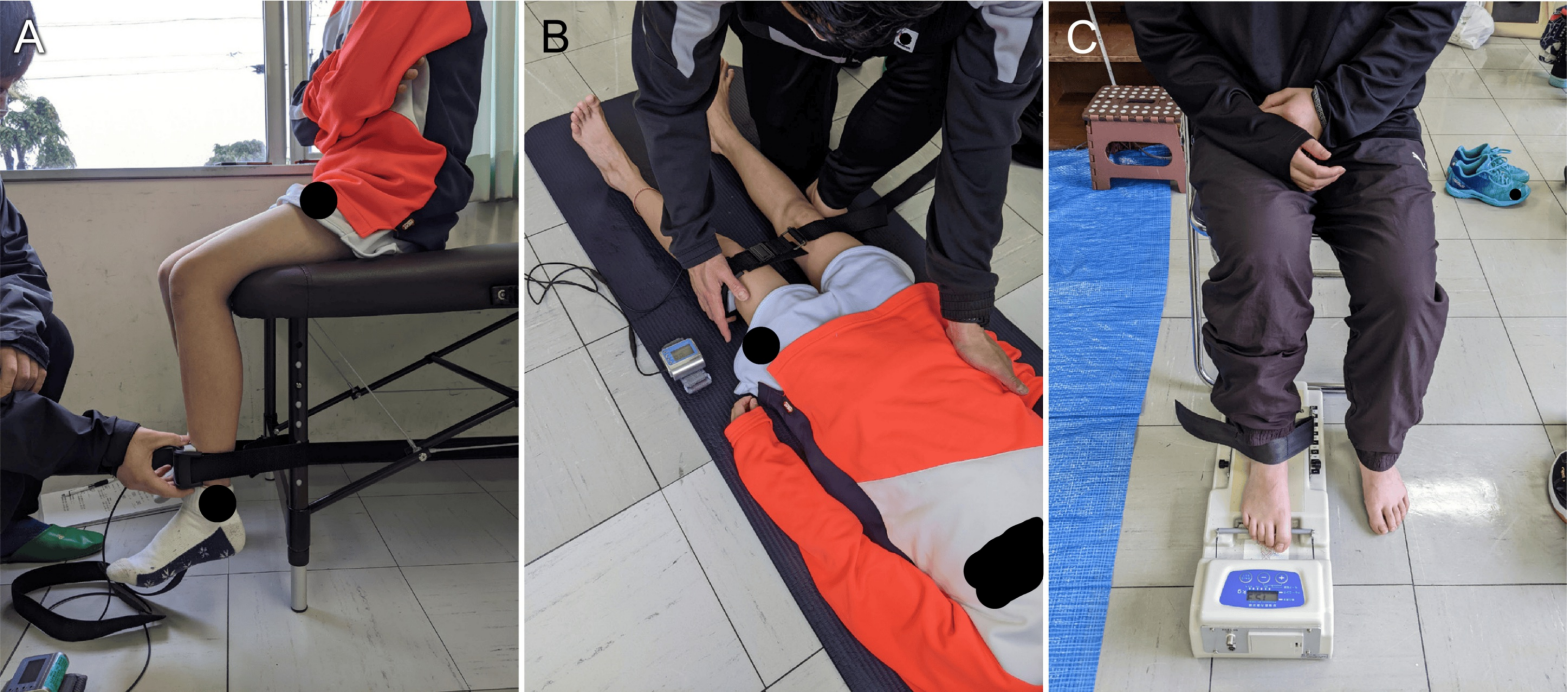
Measurements of muscle strength A. Knee extensors. B. Hip abductors. C. Toe grip

Lower limb muscle strength was measured for the hip abductors, knee extensors, and toe grip strength. These factors are involved in ankle sprains and balance function [11,18,32-38]. The strength of the hip abductors and knee extensors during maximal isometric contraction was measured using a handheld dynamometer (#μTas F-1; Anima Co., Ltd., Tokyo, Japan). Based on a previous study [39], knee extensor strength was measured in the sitting position, and hip abductor strength was assessed in the supine position (Figures 4A, 4B). The participants were instructed to exert maximal effort for one to two seconds. Adequate rest was maintained between measurements, and each test was conducted twice to calculate the average value for each side. The distance from the end of the handheld dynamometer to the joint axis was measured using a measuring tape, and the moment values were normalized by the body weight. Toe grip strength was measured using a toe grip strength meter (Takei, Japan) (Figure 4C). Thereafter, the participants were positioned in a sitting position with their knees flexed at 90° during the measurement. Before measurement, the distal phalanges of the big and fifth toes and the middle phalanges of the second to the fifth toes were adjusted to rest on the toe grip bar for maximum grip. After sufficient practice, measurements were performed twice on each side, and the average value for each side was calculated.

**Figure 5:**
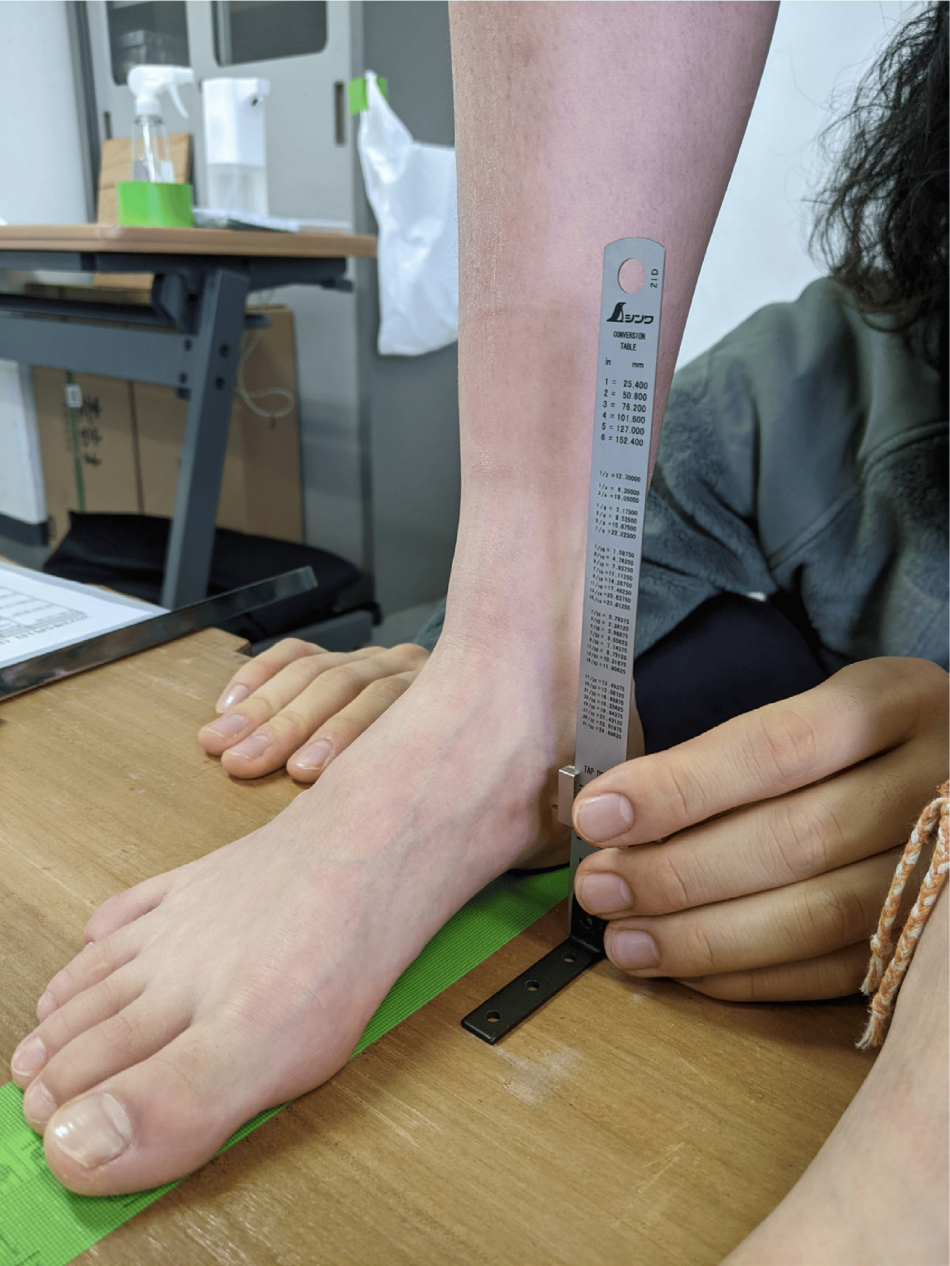
The arch-height ratio

Foot arch was assessed using the arch-height ratio [40]. The arch-height ratio primarily represents the height of the medial longitudinal arch of the foot. A high arch-height ratio indicates the presence of a well-formed medial longitudinal arch, whereas a low ratio suggests a potential deformity in its formation. The arch-height ratio was calculated by measuring the vertical distance from the tuberosity of the navicular bone to the floor in a resting standing position, and the resulting value was divided by the foot length to obtain the average value for both sides (Figure 5).

Statistical analysis

Statistical analysis was performed using IBM SPSS Statistics version 27 (IBM Corp., Armonk, NY). First, the number and percentage of participants with and without a history of ankle sprain were calculated. Participants whose dominant foot matched the sprained side were included in the analysis, with those with a sprain history categorized into the sprain group and those without a history into the non-sprain group. Statistical analysis was performed using the Shapiro-Wilk test to confirm the normality of each measurement item. To compare the sprain and non-sprain groups, the following analyses were performed. Because this study included both men and women, we first evaluated sex differences within the sprain and non-sprain groups using an unpaired t-test. The ability to squat was analyzed using the chi-squared test. An unpaired t-test was used to analyze normally distributed data, whereas the Mann-Whitney U test was used for items without normal distribution. In addition, a paired t-test was performed for normally distributed items to compare the dominant and non-dominant legs, and a Wilcoxon signed-rank test was used to analyze items in which normality was not confirmed. A p-value <0.05 was considered statistically significant. The examiner was blinded to whether each participant had a history of ankle sprain.

Ethical considerations

The participants and their guardians were informed verbally and in writing regarding the priority of protecting the participants’ rights, freedom to participate or withdraw, the study's content, and any potential effect of the study activities on their bodies. Measurements were conducted only after informed consent was obtained from the participants. This study was performed in accordance with the ethical guidelines of the 1975 Declaration of Helsinki and was approved by Kyorin University Faculty of Health Sciences Ethics Committee (approval no: 2021-65).

## Results

Table 1 presents the fundamental characteristics of participants in the sprain and non-sprain groups. Fifteen (23.4%) participants were included in the sprain group and 49 (76.6%) in the non-sprain group. All individuals in the sprain group had sustained a single LAS, with all injuries occurring more than three months before enrollment. Injuries occurred three months to one year before the study in seven participants, two years before the study in three participants, and three years before the study in one participant. The diagnosis varied, and the method used for diagnosis could not be confirmed. At the time of participation, all individuals were able to play badminton without difficulty. The non-sprain group had no history of sprain. In the sprain group, 11 (73.3%) participants had a right-dominant foot and four had a left-dominant foot. In addition, 11 (73.3%) sprained their dominant foot, two (13.3%) sprained their non-dominant foot, and two (13.3%) sprained both feet. Thus, 11 participants were included in the analysis.

**Table 1:** Basic characteristics of patients in the sprain and non-sprain groups Participants whose dominant foot matched the sprained side were included in the analysis. Those with a history of sprains were categorized into the sprain group, and those without a history were categorized into the non-sprain group. Because some expected cell counts were less than 5, Fisher’s exact test was used. No significant difference in sex distribution was observed between the sprain and non-sprain groups (p=0.533). An unpaired t-test was used to analyze normally distributed data, whereas the Mann–Whitney U test was used for items without normal distribution SD: standard deviation; BMI: body mass index

Characteristics	Sprain group (18.3%)	Non-sprain group (81.7%)	P-value
	(n=11)	(n=49)	
Sex, male, n (%)	4 (36%)	20 (40.8%)	
Sex, female, n (%)	7 (64%)	29 (59.2%)	
Height, cm, mean ± SD	145.0 ± 4.1	142.4 ± 8.4	0.15
Weight, kg, mean ± SD	36.3 ± 5.2	34.7 ± 6.3	0.27
BMI, kg/m^2^, mean ± SD	17.2 ± 1.7	17.0 ± 1.9	0.34
Age, years, mean ± SD	10.7 ± 0.7	10.7 ± 0.7	0.75
Competitive years in badminton, mean ± SD	5.0 ± 2.1	4.4 ± 1.8	0.29

Regarding the measurement data, the Shapiro-Wilk test confirmed normality for height, mSEBT, standing forward flexion, hip abductor strength, and knee extension strength. No significant differences were observed between the groups in terms of sex, height, weight, BMI, age, or years of competitive experience in badminton. Additionally, analysis of sex differences within groups showed that, in the non-sprain group, only standing forward flexion was significantly greater in females; no other measures differed by sex. The results of each measurement are listed in Table 2.

**Table 2:** Results of each measurement item ^a^Data are presented as means ± standard deviation; ^b^t-value, degrees of freedom are 58; ^c^u-value The ability to squat was analyzed using the chi-squared test. An unpaired t-test was used to analyze normally distributed data, whereas the Mann–Whitney U test was used for items without normal distribution. In addition, a paired t-test was performed for normally distributed items to compare the dominant and non-dominant legs, and a Wilcoxon signed-rank test was used to analyze items in which normality was not confirmed

Measurement item	Sprain group^a^	Non-sprain group^a^	Mean difference ± SE	Test statistic	P-value	Hedges’ g
	(n=11)	(n=49)				
Dominant foot mSEBT (%）	80.23 ± 6.18	80.50 ± 6.18	-0.27 ± 2.06	-0.13^b^	0.15	0.04
Non-dominant foot mSEBT (%）	81.54 ± 6.57	80.38 ± 7.27	1.16 ± 2.39	0.49^b^	0.90	0.16
Standing forward flexion (cm)	1.91 ± 7.50	5.19 ± 8.27	-3.28 ± 2.72	-1.21^b^	0.23	0.40
Dominant foot heel-to-buttock distance (cm)	0.27 ± 0.90	0.25 ± 0.99	0.02 ± 22.63	262.50^c^	0.76	0.03
Non-dominant foot heel-to-buttock distance (cm）	0.00 ± 0.00	0.16 ± 0.83	-0.16 ± 16.28	280.50^c^	0.50	0.21
Dominant foot hip abductor strength (Nm/kg)	0.11 ± 0.02	0.11 ± 0.02	0.00 ± 0.01	0.09^b^	0.93	0.07
Non-dominant foot hip abductor strength (Nm/kg)	0.11 ± 0.02	0.11 ± 0.02	0.00 ± 0.01	-0.06^b^	0.96	0.08
Dominant foot knee extensor strength (Nm/kg)	0.14 ± 0.04	0.11 ± 0.04	0.03 ± 0.01	2.06^b^	0.04	0.07
Non-dominant foot knee extensor strength (Nm/kg)	0.14 ± 0.04	0.11 ± 0.04	0.03 ± 0.01	2.29^b^	0.03	0.08
Dominant foot ankle dorsiflexion range of motion (°)	15.36 ± 3.41	20.20 ± 6.35	-4.84 ± 51.01	379.00^c^	0.03	0.80
Non-dominant foot ankle dorsiflexion range of motion (°)	15.55 ± 3.64	20.16 ± 6.32	-4.61 ± 51.25	388.50^c^	0.02	0.77
Dominant foot arch height ratio	13.57 ± 1.93	13.91 ± 2.12	-0.34 ± 52.34	287.50^c^	0.73	0.16
Non-dominant foot arch height ratio	14.01 ± 1.98	13.89 ± 4.39	0.12 ± 52.34	236.00^c^	0.52	0.03
Dominant foot toe grip strength	13.11 ± 4.52	13.20 ± 3.54	-0.09 ± 52.34	273.00^c^	0.95	0.03
Non-dominant foot toe grip strength	13.59 ± 3.51	12.91 ± 3.54	0.68 ± 52.34	232.50^c^	0.48	0.19

Table 3 presents the results of the squat-down tasks. The proportion of participants who successfully completed all tasks was significantly higher in the non-sprain group compared with the sprain group (45% vs. 9%; p=0.04). In contrast, the proportion of participants unable to complete at least one task was significantly higher in the sprain group than in the non-sprain group (91% vs. 55%; p=0.04). In addition, knee extension strength was significantly greater in the sprain group (dominant foot: 0.14 ± 0.04, non-dominant foot: 0.14 ± 0.04) than in the non-sprain group (dominant foot: 0.11 ± 0.04, non-dominant foot: 0.11 ± 0.04) for both feet. The ROM for ankle dorsiflexion was significantly smaller in the sprain group (dominant foot: 15.36 ± 3.41, non-dominant foot: 15.55 ± 3.64) than in the non-sprain group (dominant foot: 20.20 ± 6.35, non-dominant foot: 20.16 ± 6.32) for both feet. Notably, no significant differences were found in any of the measured items between the dominant and non-dominant feet in both groups.

**Table 3:** Ability to squat A score of 0/3 was assigned if none were successful; 1/3 if only task “keeping the arms forward” was successful; 2/3 if tasks “keeping the arms forward” and “crossing the arms in front of the chest” were successful; and 3/3 if all tasks were successful

Squat performance score	Sprain group, n (%)	Non-sprain group, n (%)
0/3	1 (9%)	9 (18%)
1/3	2 (18%)	4 (8%)
2/3	7 (63%)	14 (28%)
3/3	1 (9%)	22 (45%)

## Discussion

The results of this study indicated that the sprain group had a higher proportion of individuals who were unable to squat, a greater knee extension strength, and a smaller ankle dorsiflexion ROM. Fifteen (six males, nine females) participants (10-12 years) had a history of ankle sprains. A study found that the incidence of ankle sprains was higher in children (0-12 years) than in adolescents (13-17 years) and adults (18 years and older), and higher in females than in males [32]. Furthermore, we found a higher proportion of females with ankle sprains, confirming a similar trend. Among elementary school badminton players aged 7-12 years in Japan, 14.9% reported having an ankle injury in the past 12 months [16]. In comparison, the present study found a slightly higher rate (23.4%). This discrepancy may be attributed to the fact that the previous study included 7-12-year-old badminton players, whereas this study included those aged 10-12 years. Therefore, as age increases, the intensity of competition increases, making injuries more likely to occur [32], which may explain the higher incidence of sprains.

Previous studies comparing the dominant and non-dominant feet have suggested potential differences in physical function and movement strategies between the two sides [41,42]. Because badminton is an asymmetric sport, the mechanism of injury may differ between the dominant and non-dominant sides. Although there are no studies on sprains, injuries to the anterior cruciate ligament tend to occur more frequently in the knee opposite to the hand holding the racket when landing on one leg during backhand overhead strokes [43]. However, during side-step cuts and backward steps, there is a high rate of knee injury to the hand side of racket handlers [43]. These findings suggest that limb dominance and stroke mechanics may influence the loading patterns and mechanisms leading to lower limb injuries in badminton. Therefore, distinguishing between the dominant and non-dominant limbs is important when analyzing injury mechanisms or developing preventive strategies.

This study focused on individuals whose injured side matched their dominant foot, and 11 participants met this criterion. Sports often involve asymmetrical movements, leading to different roles for the dominant and non-dominant feet. In badminton, the cross-sectional area, width, and thickness of the patellar and Achilles tendons are larger in the dominant foot [44]. Besides, injuries in badminton predominantly occur in the lumbar spine, knee joints, and shoulder joints on the dominant side [10]. Therefore, it is necessary to separately consider the dominant and non-dominant feet. As a result, this study included individuals who sprained their dominant foot, which is often involved in lunging. The percentage of sprains occurring on the dominant foot was 73.3%, which was significantly higher than that on the non-dominant foot (13.3%) and both feet (13.3%), indicating a higher risk of spraining the dominant foot.

Furthermore, in badminton, the force applied to the shoes tends to be directed towards the front and anterolateral sides on the racket side (dominant foot side), whereas it tends to be directed towards the lateral and posterolateral sides on the non-racket side [45]. Although the injury mechanism of sprains is not yet clearly understood, rapid inversion and internal rotation during the initial ground contact may be contributing factors [46]. This implies that situations in which force is applied to the anterolateral part of the shoe are expected. Because an analysis of sprain movements was not conducted in this study, these results only demonstrate the relationship with the history of sprains. Further research on movement analysis and longitudinal observation of the occurrence of sprains is warranted.

An adequate ankle dorsiflexion ROM is necessary for complete squatting. Following a sprain, there is restricted dorsiflexion ROM on the injured side [47-49]. In this study, the sprain group exhibited limited dorsiflexion ROM, which is consistent with the results of previous studies. Some studies have suggested that a small ankle dorsiflexion ROM is associated with a higher risk of sprains and predicts LAS [50-52]. Postural sway and proprioception have been considered other predictors of LAS, but it remains inconclusive [50]. In a systematic review and meta-analysis of sex differences in risk factors for sprains, the risk factors for men included previous sprains, high BMI, high body weight, low isometric hip abductor strength, and poor dynamic balance ability [53].

Decreased dorsiflexor muscle strength is a risk factor for sprain in women [53]. Notably, the ankle dorsiflexion ROM was not reported as a risk factor in that study. Thus, it remains unclear whether limited ankle dorsiflexion ROM is a risk factor; however, it appears to be a consequence of sprain. An interesting finding of this study was that the ankle dorsiflexion ROM of the dominant and non-dominant feet in the sprain group was significantly smaller than that in the non-sprain group. Therefore, participants with a small dorsiflexion ROM of the ankle joint may have sustained a sprain. Hence, a longitudinal study investigating the changes in dorsiflexion ROM of the sprained ankle joint is warranted.

Furthermore, the sprain group exhibited stronger knee extension strength. Restricted ankle dorsiflexion leads to reduced ROM for knee flexion during landing in lunging, resulting in increased ground reaction forces [54]. This means that a greater knee extension strength is required to counteract the reaction forces on landing. In this study, the sprain group had restrictions in ankle dorsiflexion. Therefore, during landing while playing badminton, participants in the sprain group required more knee extension strength than did the participants in the non-sprain group, demonstrating stronger knee extension strength in the sprain group. Furthermore, the comparison of knee extension strength across all participants showed no significant differences between the dominant and non-dominant feet. Therefore, these results can be interpreted as characteristics specific to the sprain group. However, the effect size of knee extensor muscle strength was very small, although significant differences were observed. Therefore, the results on knee extension muscle strength should be cautiously interpreted. Motion analysis of badminton movements was not performed in this study. Future investigations should specifically examine lunge mechanics to clarify their relationship with knee extension strength.

This study has some limitations. First, as this was a cross-sectional study, it could not address the causal relationship between a history of ankle sprain and physical characteristics. Therefore, further longitudinal investigations are required. Second, the history of ankle sprain was assessed using a questionnaire, which limited the ability to evaluate the specifics of the injury circumstances. Thus, future studies should collate data on where and how ankle sprains occur in badminton courts. Third, this study was limited to participants aged 10-12 years; therefore, findings related to other age groups remain unclear. The risk of ankle sprain varies according to age group [55]; consequently, it is important to broaden the study population and include data from adolescents, who represent a significant proportion of the competitive population. Fourth, our sample size was small. The injury rate of sprains in elementary school badminton players is 14.9% [29]. Ideally, the non-sprain and sprain groups should have an equal number of participants. However, based on the injury rate, it is difficult to collect a sufficient number of players with a history of sprain. When the effect size was calculated owing to the small sample size, only the ankle dorsiflexion ROM was larger (0.77-0.80). Therefore, we believe that ankle dorsiflexion ROM provides meaningful data regarding previous sprains.

In badminton, repetitive lunge and jumping movements result in a high risk of ankle sprain, which is the most common type of lower limb injury. Limited ankle dorsiflexion range of motion was identified as a characteristic of the sprain group, suggesting it is a critical factor requiring special attention after a sprain.

## Conclusions

This study examined the relationship between ankle sprain history and the physical abilities of elementary school badminton players. Participants with a prior sprain showed a higher incidence of difficulty performing squats, greater knee extension strength, and reduced ankle dorsiflexion ROM. Although several foot-related measures were implicated, knee extension strength also played a role. Given the large effect size for ankle dorsiflexion ROM and the small effect size for knee extension strength, ankle dorsiflexion ROM appears to be the measure most influenced by sprain history. Although the study focused on individuals who sprained their dominant foot, no differences were observed between the dominant and non-dominant feet. Further research is needed to determine whether these characteristics are typical of early childhood or specific to badminton. Additionally, it is important to clarify the details of events related to ankle sprain injury and study the differences in movement patterns based on the history of sprain through motion analysis.

## References

[1] (2024). Badminton World Federation: about badminton. https://corporate.bwfbadminton.com/about/about-badminton/.

[2] (2024). Nippon Badminton Association: registered members. https://www.badminton.or.jp/nba/regist.html.

[3] Low JFL, Mohamad NI, Ong KB, Aziz SA, Abdullah MR, Maliki ABHM (2017). The developmental pathways of Malaysian elite youth badminton players. J Fundam Appl Sci.

[4] Engebretsen L, Soligard T, Steffen K (2013). Sports injuries and illnesses during the London Summer Olympic Games 2012. Br J Sports Med.

[5] Høy K, Lindblad BE, Terkelsen CJ, Helleland HE, Terkelsen CJ (1994). Badminton injuries--a prospective epidemiological and socioeconomic study. Br J Sports Med.

[6] Jørgensen U, Winge S (1987). Epidemiology of badminton injuries. Int J Sports Med.

[7] Jørgensen U, Winge S (1990). Injuries in badminton. Sports Med.

[8] Shariff AH, George J, Ramlan AA (2009). Musculoskeletal injuries among Malaysian badminton players. Singapore Med J.

[9] Guermont H, Le Van P, Marcelli C, Reboursière E, Drigny J (2021). Epidemiology of injuries in elite badminton players: a prospective study. Clin J Sport Med.

[10] Miyake E, Yatsunami M, Kurabayashi J (2016). A prospective epidemiological study of injuries in Japanese national tournament-level badminton players from junior high school to university. Asian J Sports Med.

[11] Earl JE, Hertel J (2001). Lower-extremity muscle activation during the star excursion balance tests. J Sport Rehabil.

[12] Marchena-Rodriguez A, Cabello-Manrique D, Ortega-Avila AB, Martinez-Rico M, Cervera-Garvi P, Gijon-Nogueron G (2024). Aetiology, epidemiology and treatment of musculoskeletal injuries in badminton players: a systematic review and meta-analysis. Res Sports Med.

[13] Hensley LD, Paup DC (1979). A survey of badminton injuries. Br J Sports Med.

[14] Krøner K, Schmidt SA, Nielsen AB, Yde J, Jakobsen BW, Møller-Madsen B, Jensen J (1990). Badminton injuries. Br J Sports Med.

[15] Cronin J, McNair PJ, Marshall RN (2003). Lunge performance and its determinants. J Sports Sci.

[16] Phomsoupha M, Laffaye G (2015). The science of badminton: game characteristics, anthropometry, physiology, visual fitness and biomechanics. Sports Med.

[17] Fong DT, Mok KM, Thompson IM, Wang Y, Shan W, King MA (2023). A lateral ankle sprain during a lateral backward step in badminton: a case report of a televised injury incident. J Sport Health Sci.

[18] Friel K, McLean N, Myers C, Caceres M (2006). Ipsilateral hip abductor weakness after inversion ankle sprain. J Athl Train.

[19] Gerber JP, Williams GN, Scoville CR, Arciero RA, Taylor DC (1998). Persistent disability associated with ankle sprains: a prospective examination of an athletic population. Foot Ankle Int.

[20] Bagehorn T, de Zee M, Fong DT, Thorborg K, Kersting UG, Lysdal FG (2024). Lateral ankle joint injuries in indoor and court sports: A systematic video analysis of 445 nonconsecutive case series. Am J Sports Med.

[21] Fong DT, Hong Y, Chan LK, Yung PS, Chan KM (2007). A systematic review on ankle injury and ankle sprain in sports. Sports Med.

[22] Grimandi R, Tissier F, Andro C, Tardy D, Gunepin FX, Rannou F, Giroux-Metges MA (2022). The hamstrings are more impacted than the quadriceps after severe ankle sprain. Medicine (Baltimore).

[23] Yeung MS, Chan KM, So CH, Yuan WY (1994). An epidemiological survey on ankle sprain. Br J Sports Med.

[24] Cheng WL, Jaafar Z (2020). Effects of lateral ankle sprain on range of motion, strength and postural balance in competitive basketball players: a cross-sectional study. J Sports Med Phys Fitness.

[25] Taketomi S, Kawaguchi K, Mizutani Y (2024). Factors associated with a lateral ankle sprain in young female soccer players: a prospective cohort study. Orthop J Sports Med.

[26] Wang L, Yu G, Zhang X, Wang YZ, Chen YP (2023). Relationship between ankle pain, range of motion, strength and balance in individuals with functional ankle instability: a cross-sectional study. BMC Musculoskelet Disord.

[27] Gabriner ML, Houston MN, Kirby JL, Hoch MC (2015). Contributing factors to star excursion balance test performance in individuals with chronic ankle instability. Gait Posture.

[28] Matsumura M, Fujimoto S, Kurihara Y (2022). Injury characteristics of elementary school badminton players: cross-sectional study using a questionnaire (in Japanese). Rigakuryoho Kagaku.

[29] Liu X, Imai K, Zhou X, Watanabe E (2022). Influence of ankle injury on subsequent ankle, knee, and shoulder injuries in competitive badminton players younger than 13 years. Orthop J Sports Med.

[30] Plisky PJ, Rauh MJ, Kaminski TW, Underwood FB (2006). Star Excursion Balance Test as a predictor of lower extremity injury in high school basketball players. J Orthop Sports Phys Ther.

[31] Butler RJ, Southers C, Gorman PP, Kiesel KB, Plisky PJ (2012). Differences in soccer players' dynamic balance across levels of competition. J Athl Train.

[32] Ambegaonkar JP, Mettinger LM, Caswell SV, Burtt A, Cortes N (2014). Relationships between core endurance, hip strength, and balance in collegiate female athletes. Int J Sports Phys Ther.

[33] Gribble PA, Robinson RH (2009). An examination of ankle, knee, and hip torque production in individuals with chronic ankle instability. J Strength Cond Res.

[34] Kwon YU (2023). Lower extremity muscle activation during the Star Excursion Balance Test in patients with chronic ankle instability and copers. Medicina (Kaunas).

[35] Norris B, Trudelle-Jackson E (2011). Hip- and thigh-muscle activation during the star excursion balance test. J Sport Rehabil.

[36] Quinlan S, Fong Yan A, Sinclair P, Hunt A (2020). The evidence for improving balance by strengthening the toe flexor muscles: a systematic review. Gait Posture.

[37] Spink MJ, Fotoohabadi MR, Wee E, Hill KD, Lord SR, Menz HB (2011). Foot and ankle strength, range of motion, posture, and deformity are associated with balance and functional ability in older adults. Arch Phys Med Rehabil.

[38] Váczi M, Ambrus M (2014). Chronic ankle instability impairs quadriceps femoris contractility and it is associated with reduced stretch-shortening cycle function. Isokinet Exerc Sci.

[39] Katoh M, Yamasaki H (2009). Comparison of reliability of isometric leg muscle strength measurements made using a hand-held dynamometer with and without a restraining belt. J Phys Ther Sci.

[40] Waseda A, Suda Y, Inokuchi S, Nishiwaki Y, Toyama Y (2014). Standard growth of the foot arch in childhood and adolescence--derived from the measurement results of 10,155 children. Foot Ankle Surg.

[41] Nakahira Y, Taketomi S, Kawaguchi K (2022). Kinematic differences between the dominant and nondominant legs during a single-leg drop vertical jump in female soccer players. Am J Sports Med.

[42] Paillard T, Noé F (2020). Does monopedal postural balance differ between the dominant leg and the non-dominant leg? A review. Hum Mov Sci.

[43] Kimura Y, Ishibashi Y, Tsuda E, Yamamoto Y, Tsukada H, Toh S (2010). Mechanisms for anterior cruciate ligament injuries in badminton. Br J Sports Med.

[44] Bravo-Sánchez A, Abián P, Jiménez F, Abián-Vicén J (2019). Myotendinous asymmetries derived from the prolonged practice of badminton in professional players. PLoS One.

[45] Smith N, Lees A (1994). An ergonomic evaluation of the shoe-surface interface in badminton. Science and Racket Sports I.

[46] Wagemans J, Bleakley C, Taeymans J, Kuppens K, Schurz AP, Baur H, Vissers D (2023). Rehabilitation strategies for lateral ankle sprain do not reflect established mechanisms of re-injury: a systematic review. Phys Ther Sport.

[47] Cady KP, De Ste Croix M, Deighan M (2024). Effect of sex and lateral ankle sprain history on dorsiflexion range of motion asymmetry during the weight bearing lunge test. Int J Sports Phys Ther.

[48] Miller H, Fawcett L, Rushton A (2020). Does gender and ankle injury history affect weightbearing dorsiflexion in elite artistic gymnasts?. Phys Ther Sport.

[49] Senanayake S, Premakumara T, Kodagoda P, Jayasekara H (2021). Influence of weight-bearing dorsiflexion (WBDF) on ankle injury history among semi-professional recreational basketball players. Adv J Grad Res.

[50] de Noronha M, Refshauge KM, Herbert RD, Kilbreath SL, Hertel J (2006). Do voluntary strength, proprioception, range of motion, or postural sway predict occurrence of lateral ankle sprain?. Br J Sports Med.

[51] Pope R, Herbert R, Kirwan J (1998). Effects of ankle dorsiflexion range and pre-exercise calf muscle stretching on injury risk in army recruits. Aust J Physiother.

[52] Willems TM, Witvrouw E, Delbaere K, Mahieu N, De Bourdeaudhuij I, De Clercq D (2005). Intrinsic risk factors for inversion ankle sprains in male subjects: a prospective study. Am J Sports Med.

[53] Mason J, Kniewasser C, Hollander K, Zech A (2022). Intrinsic risk factors for ankle sprain differ between male and female athletes: a systematic review and meta-analysis. Sports Med Open.

[54] Fong CM, Blackburn JT, Norcross MF, McGrath M, Padua DA (2011). Ankle-dorsiflexion range of motion and landing biomechanics. J Athl Train.

[55] Doherty C, Delahunt E, Caulfield B, Hertel J, Ryan J, Bleakley C (2014). The incidence and prevalence of ankle sprain injury: a systematic review and meta-analysis of prospective epidemiological studies. Sports Med.

